# Retraction: Integrative analysis of serum microorganisms and serum metabolomics in osteoporosis patients based on 16S rDNA sequencing and UHPLC/MS-based metabolomics

**DOI:** 10.3389/fmed.2026.1937107

**Published:** 2026-07-16

**Authors:** Frontiers Editorial Office

The journal and Chief Editors retract the 24 September 2025 article cited above.

Following publication, Frontiers Research Integrity Auditing team identified concerns regarding the validity of the data in the article. The authors failed to provide the raw data or a satisfactory explanation during the investigation, which was conducted in accordance with Frontiers' policies. Given the concerns, and the lack of raw data, the editors no longer have confidence in the findings presented in the article.

This retraction was approved by the Chief Editors of Frontiers in Medicine and the Chief Executive Editor of Frontiers. The authors have not responded to any correspondence regarding this retraction.

